# The impact of improper promotion of sports lottery on the harm of purchasing sports lottery: an intermediary in a regulatory chain

**DOI:** 10.3389/fpsyt.2025.1670697

**Published:** 2026-02-04

**Authors:** Gai Li, Yang Li, Lian Liu

**Affiliations:** 1 School of Physical Education, Central China Normal University, Wuhan, School of Physical Education, Wuhan, Hubei, China; 2 School of Physical Education, Huzhou University, Huzhou, Zhejiang, China

**Keywords:** sports lottery players, inappropriate publicity exposure, obsessive passion of lottery, problem lottery playing, lottery playing harm, responsible betting beliefs

## Abstract

**Introduction:**

To explore the relationship between the improper promotion of lottery games and the harm caused by lottery players' purchase of lottery tickets, as well as the chain mediating role of the compulsive tendency of lottery purchase addiction, bad lottery purchase behaviors, and responsible lottery purchase beliefs.

**Methods:**

A questionnaire survey was conducted on 600 lottery players, and the data were analyzed using SPSS 27.0 and the PROCESS plugin.

**Results:**

(1) The improper promotion of sports betting is directly related to the harm caused by sports betting; (2) The improper promotion of sports betting is related to the harm through three indirect pathways: namely, through the compulsive tendency of gambling addiction, bad gambling behaviors, and the chain relationship between gambling addiction and bad gambling behaviors as mediating factors; (3) The belief of responsibility when purchasing lottery tickets played a moderating role in the impact of improper sports betting promotion on gambling addiction behavior, which could buffer the impact of improper sports betting promotion on gambling addiction behavior and the impact of problem gambling behavior on the harm of lottery betting. The responsible purchase belief also played a moderating role in the impact of improper lottery betting promotion on gambling addiction behavior, which could buffer the impact of improper lottery betting promotion on gambling addiction behavior and the impact of problem gambling behavior on the harm of lottery betting.

**Conclusion:**

The mediating model constructed in this study to some extent reveals the mechanism of the impact of improper promotion on the harm of sports betting, and provides theoretical support and practical guidance for preventing the risk of harm from sports betting.

## 1 Introduction

In recent years, along with the rapid growth of the global gambling industry, the sports lottery industry has also been booming. According to incomplete statistics, the number of Chinese lottery players has expanded to 300 million (1). Rational purchase of lottery tickets by Sports lottery player can satisfy their own psychological needs and, at the same time, increase the state’s financial income and promote the development of sports and health, and social welfare to a certain extent (2). However, irrational lottery purchasing can produce a series of harms, such as physical and mental health harms (insomnia, anxiety) (3), financial harms (suffering economic losses) (4), interpersonal harms (interpersonal communication barriers or conflicts), work or study harms (reduced performance), cultural harms (experiencing social isolation), social harms (crime), and so on. Therefore, exploring the risk factors for the formation of betting harms among sports bettors and formulating effective preventive strategies are key issues that need to be addressed for the healthy and sustainable development of the sports lottery industry.

Currently, some scholars have also explored the risk factors of betting hazards, which involve individual early betting experience, cognitive distortion (5), pecuniary motives, inappropriate publicity for betting, and peer attitudes toward purchasing bets, etc. Among them, the risk factors of inappropriate publicity for sports betting have been discussed. Among them, the research on inappropriate publicity of sports betting is more common; for example explored the mechanism of the influence of inappropriate publicity of sports betting on problematic betting based on the pure exposure effect and the stimulus-organism-response theory (6) but few studies have examined the mechanism of inappropriate publicity affecting the harm of problematic betting. Second, there may be other individual risk factors (e.g., obsessive passion, lottery purchase behavior) in the path of inappropriate publicity exposure affecting lottery purchase harm. Research shows a positive correlation between obsessive passion for lottery purchases and the severity of the Lottery playing harms (7). Lottery buyers with high participation and preference for instant prize gratification, such as instant winners, are at higher risk of experiencing gambling harm (8). In addition, based on the risk-protection integration theory of gambling, protective factors can buffer the harm caused by risk factors. Responsible gambling as a preventive strategy has been focused on by many theoretical researchers as well as practical operators; e.g., studies have shown that responsible gambling influences gambling behavior and can minimize gambling harms (9). Individuals who hold responsible gambling beliefs will gamble under rational gambling cognition, such as by boycotting gambling marketing advertisements to reduce the risk of their problem gambling (10). Therefore, sports bettors’ responsible betting beliefs may buffer the harms of betting caused by the inappropriate publicity exposure of sports betting.

In summary, this study intends to construct a chain mediation model with adjustments to explore the effects of inappropriate publicity on the harm of betting purchases, as well as the mediating roles of obsessive passion for betting purchases and problematic betting behaviors and the moderating roles of responsible betting purchasing beliefs, to provide theoretical support and a practical basis for the mitigation of betting purchasing harms and the prevention of problematic bettors.

## 2 Literature review and research hypotheses

### 2.1 The effect of sports lottery inappropriate publicity exposure on the harm of purchasing lottery tickets

Sports lottery Inappropriate publicity exposure (11) refers to the false, misleading, and deceptive publicity conducted by various communication subjects of the sports lottery. As a distal risk factor of the harm of betting, the inappropriate publicity exposure of sports lottery may bring the harm of betting to sports lottery players (4) for the following reasons: On the one hand, the proliferation of inappropriate publicity exposure expands the scope of the harm of betting for sports lottery players and increases the risk of irrational betting. It has been found that there is a correlation between the severity of betting harms and exposure to betting advertisements and betting promotions, and the more sports bettors are exposed to inappropriate publicity information, the more Negative mood, health problems, and social harms are associated with betting (12). Some scholars have also pointed out that inappropriate publicity as an inducement can increase bettors’ betting expenditures, length of betting participation, and frequency of betting and lead to betting harms; it also makes it more difficult for gamblers to exercise self-control over gambling (13). Some people who have higher click-through and recall rates of inappropriately publicized advertisements for sports betting also experience more harm from betting purchases, especially those who are undergoing treatment for problematic betting and suffer more or bring more harm because it is more difficult for them to resist the attraction of the publicity (14). On the other hand, the improper propaganda of sports betting can lead to an unhealthy betting psychology of sports bettors, which in turn brings harm. For example, the main body of communication uses various means and channels of communication to carry out a large number of bad communications, which strengthens the stimulation of the unhealthy psychology of lottery players (flopping mentality, illusion of control, gambler’s fallacy, superstition, speculative mentality, and materialism) (15). These falsehoods and exaggerated publicity content will produce psychological inducement to sports lottery players, making them ignore the risks of purchasing lottery tickets and reduce rational thinking in the process of purchasing lottery tickets, thus causing physical and mental harm to lottery players (16). Based on this, hypothesis H1: Inappropriate publicity exposure significantly positively predicts the harm of lottery players’ lottery purchase.

### 2.2 The possible mediating role of obsessive passion in the exposure of inappropriate publicity exposure in the harm of lottery purchase

Obsessive passion is a psychological state in which Sports lottery player are unable to control their impulse to buy lottery tickets and buy lottery tickets irrationally (J. 17). Obsessive passion may play a mediating role between the harms of sports betting publicity exposure and the harms of betting. On the one hand, inappropriate publicity of sports betting can lead to obsessive passion of sports bettors. Sports lottery advertisements serve as a possible environmental cue to elicit an individual’s impulse to purchase lottery tickets (18). The reason for this is that inappropriate sports lottery publicity advertisements, especially those that are easy to win and winners will break out, all directly or indirectly enhance the illusion of control of sports lottery players and, at the same time, reduce their perceived risk of purchasing the lottery, which will lead to an increase in the passion of sports lottery players to purchase the lottery, thus prompting the players to produce irrational lottery purchasing behaviors (19). On the other hand, obsessive passion for buying lottery tickets has a positive predictive effect on the harm of buying lottery tickets. Based on the dichotomy theory of passion, obsessive passion may produce some non-adaptive outcomes, such as preventing individuals from enjoying themselves during activity involvement and affecting mental health (20). Previous studies have shown (21) that obsessive-obsessive passions are associated with many negative outcomes, such as rigid adherence, negative affect, and emotional exhaustion. A recent follow-up study also showed that gambling harmony passion was not associated with problem gambling, whereas obsessive-obsessive passion was a risk factor for gambling harms, being positively associated with the severity of gambling problems and accompanying impulsivity and anxiety, doubling the risk of developing a gambling problem, and negatively associated with higher levels of well-being (7). Obsessive passion also causes rigid adherence to the problematic gambler’s gambling activities (22), leading to the development of emotions such as anxiety and guilt as well as pathological gambling dependence, which in turn leads to gambling harms. Based on this, the research hypothesis H2 is proposed: obsessive passion for lottery purchasing mediates the relationship between inappropriate publicity, positively predicting the harms of problematic lottery purchases among lottery players.

### 2.3 Possible mediating role of problematic purchasing behaviors in the harms of purchasing lottery tickets caused by inappropriate publicity exposure of sports lottery tickets

Problem buying refers to the buying behavior that has a negative impact on individuals and related others (family or friends, etc.) (23). Problematic purchases may mediate the effects of inappropriate publicity exposure on the harms of lottery purchases. On the one hand, exposure to inappropriate sports betting publicity may lead to problematic purchasing behaviors among sports bettors. First, based on the trigger effect and stimulus-response theory (24), advertising campaigns have a specific contextual nature; the individual’s subconscious mind will be stimulated by the advertising message in a certain period of time and will subconsciously expect or create a similar situation and, to a certain extent, trigger convergent behavior. Inappropriate body lottery propaganda (e.g., easy to win, jackpot, etc. information) will lead people to subconsciously expect or associate with the advertised information. Second, the priming effect (25) states that previously presented stimuli will facilitate and ease the processing of the same or similar stimuli performed later. Therefore, sports bettors who are frequently exposed to sports betting advertisements are susceptible to the priming effect, which triggers their implicit memory and then induces problematic betting purchase behavior. Some studies have shown that exposure to betting advertisements is positively correlated with the frequency of betting behavior and problematic betting (26, 27). Advertisements may trigger their thoughts about betting, increased betting intentions, and actual gambling behavior (28). A study on the association between exposure to betting advertisements and actual gambling behavior among adolescents and young adults also found (19) a strong association between exposure to betting propaganda and both general and problematic betting behaviors. Thus, inappropriate sports betting publicity can increase problematic purchasing behavior among sports bettors. On the other hand, sports bettors’ problematic betting behavior can bring about betting harms. Based on the basic view of behavioral addiction, pathological gambling behavior shows a typical developmental trajectory of addiction, in which bettors experience strong impulses that are uncontrollable, and only through continuous participation in betting activities can they obtain immediate reward feedback and then fall into a vicious cycle of “impulse-behavior-reward,” which leads to many unsuccessful withdrawal experiences, causing many adverse effects on the individual’s life (29). Problem gambling usually involves different types of gambling harms that present themselves in various ways, such as financial loss, relationship breakdown or conflict, emotional or psychological harm, diminished health, cultural harms, reduced work productivity, and criminal behavior (3, 30). From a physiological perspective, bettors who participate in betting games have neurotransmitters such as dopamine in a state of continuous activation, which makes physiological and psychological risk-taking behaviors escalate (31), which may lead to sustained harm. Based on the above analysis, the research hypothesis H3: Problematic purchasing behavior mediates the relationship between inappropriate publicity of sports betting positively predicting the harms of bettors’ purchases is proposed.

### 2.4 The effect of compulsive lottery purchasing passion on problematic lottery purchasing behavior

Lottery players’ obsessive passion for lottery purchasing can lead to problematic lottery purchasing behavior. First of all, based on the binary model of passion (20), obsessive passionate sports bettors have a stronger impulse to buy lottery tickets and will uncontrollably continue to participate in the lottery purchase activities and even be more aggressive in the purchase of lottery tickets (32), which is prone to problems. Moreover, obsessive-obsessive passions mostly arise from extrinsic betting motives (e.g., monetary motives), whether it is wanting to win after winning or wanting to get back the money if the sunk costs are too high, which can lead to guilt, stress, and a variety of negative consequences (C.-K. 33). Research has shown that obsessive passion in bettors is highly correlated with betting craving, with higher levels of arousal elevating betting craving, which in turn increases betting behavior (34). An experimental study showed that bettors who experience winning continue to have high levels of craving for betting because experiencing rare, lucky, and serendipitous events (e.g., a big win) causes individuals to maintain a perception of being personally luckier (i.e., they believe they have the skills or ability to win in a game of pure chance), which makes problematic betting behavior more severe (35). In addition, chasing losses also leads to a strong craving for betting in response to betting-related cues, and their normal internal control mechanisms do not work in response to this strong craving (36), which in turn leads to a higher level of betting during the betting period and triggers problematic betting behaviors. Based on the above analysis, the research hypothesis H4 is proposed: obsessive passion for betting and problematic betting behavior play a chain mediating role in the relationship between inappropriate publicity of sports betting positively predicting the harm of betting.

### 2.5 Possible moderating role of responsible color buying beliefs

Responsible lottery purchasing emphasizes that individuals should remain calm and self-controlled when engaging in lottery purchasing activities for healthy lottery purchasing. Previous research suggests that responsible lottery purchasing beliefs may reduce lottery purchasing harms (37). First, responsible lottery purchasing beliefs can buffer problematic lottery purchasing behaviors caused by inappropriate publicity. Studies have shown that individual cognitive bias is a proximal risk factor for problematic lottery purchases (38). Individuals with responsible gambling beliefs have correct risk perceptions of gambling and can view gambling rationally, and they tend to anticipate the risks of gambling, thus buffering cognitive biases (e.g., illusion of control, probabilistic fallacy, and interpretive bias) generated by external adverse information and thus reducing problematic gambling behavior (39). Second, responsible lottery purchase beliefs buffer the obsessive passion to purchase lottery tickets caused by inappropriate publicity of the lottery. Previous research has indicated that individuals are vulnerable to obsessive passion for betting caused by inappropriate publicity for betting (6). However, bettors with the belief of responsible betting will be responsible for their betting behavior. When faced with inappropriate publicity exposure to information about sports betting, they tend to use rational strategies such as evaluative judgments to transition from highly aroused obsessive-compulsive “hot” emotions to “cold”emotions, resulting in more standardized decision-making behaviors (40). Again, responsible gambling beliefs may moderate the harms of gambling caused by problematic gambling behaviors. Research has shown that bettors who hold responsible betting beliefs exert control over their betting behaviors by engaging in strategies that mitigate any of the various negative outcomes (e.g., Negative mood, cognitive distortions, financial losses, suicidal intent, etc.) associated with problematic betting (37). Based on the above analysis, research hypotheses H5, H6, and H7 were proposed. H5: Responsible betting beliefs moderated the relationship between inappropriate publicity for sports betting and problematic betting behaviors; H6: Responsible betting beliefs moderated the relationship between inappropriate publicity and obsessive-obsessive passions; and H7: Responsible betting beliefs moderated the relationship between inappropriate publicity and harms of betting purchases.

### 2.6 Research hypothesis

**Figure 1:**
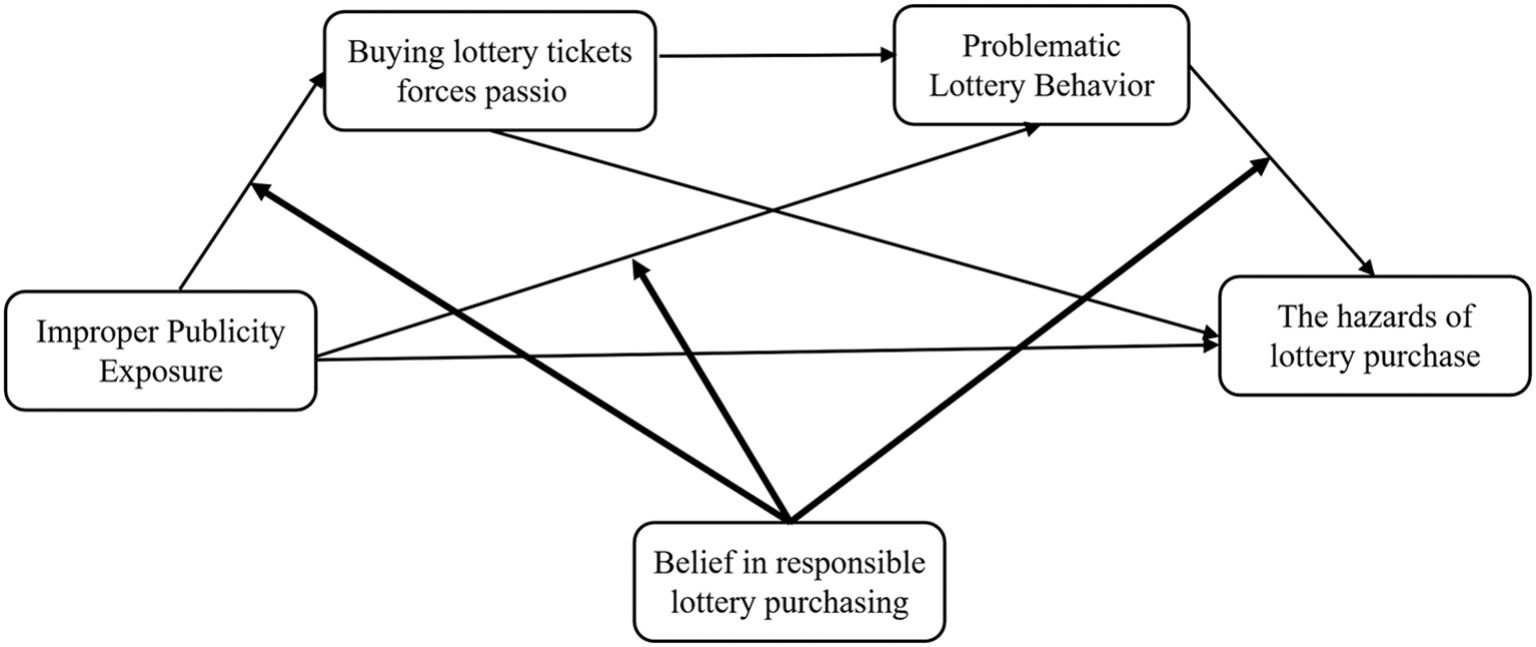
Simplified model for studying the relationship between variables.

The hypothetical model of this study is shown in **Figure 1**, which is derived from the previous theoretical analysis. Taking improper publicity exposure as the independent variable, lottery purchase harm as the dependent variable, lottery purchase obsessive passion and problem lottery purchase behavior as the mediating variables, and responsible lottery purchase belief as the moderating variable, the following simplified model diagram is obtained:

## 3 Subjects and methods

### 3.1 Subjects

Based on the stratified sampling method of geographical distribution and lottery sales levels, lottery buyers in Chongqing, Wuhan and Xiangyang of Hubei Province were selected as the research subjects. Questionnaires were distributed both online and offline, among which the online questionnaires were sent out one-to-one. The offline questionnaires were given to the lottery buyers at the sales points one-on-one. After each questionnaire was completed, the respondents were allowed to purchase three lottery tickets worth 2 RMB each or a 6 RMB red envelope. A total of 600 questionnaires were distributed, 592 were returned, and after excluding invalid questionnaires, 586 valid questionnaires were obtained. The basic information of the survey respondents is shown in **Table 1**.

**Table 1:** List of basic information of survey respondents (N = 586).

Category	Attribute	Number of persons	Percentage (%)
Gender	Male	318	54.2%
	Female	268	45.8%
Age	Below 30 years old	298	50.9%
	30–44 years old	171	29.2%
	45–59 years old	92	15.7%
	60 years old and above	25	4.3%
The level of education	Junior high school or below	62	10.6%
	High school or vocational high school	113	19.1%
	College	154	26.3%
	Bachelor’s degree	181	30.9%
	Graduate students and above	76	13.1%
Years of purchasing lottery tickets	1 year and below	207	35.2%
	2~3 years	172	29.5%
	4~6 years	93	15.9%
	7~10 years	57	9.7%
	More than 10 years	57	9.7%

### 3.2 Research tools

#### 3.2.1 Lottery purchasers questionnaire on harmful effects of lottery playing

The Hazards of Purchase Questionnaire of Sports lottery player’ Purchase Health Questionnaire prepared by Wang Bin et al. (12). was used. The sub-questionnaire has a total of 16 entries, which are divided into 5 dimensions: physical health hazards (4 entries), Negative mood hazards (3 entries), financial loss hazards (3 entries), interpersonal relationship hazards (3 entries), and work/study hazards (3 entries). The questionnaire was scored on a 4-point scale, with 0 indicating never, 1 indicating occasionally, 2 indicating often, and 3 indicating always. The reliability of the questionnaire was reported to be good (ZhangLei, 2024). In order to examine the hazards of color purchase more objectively, the frequency of the hazards was evaluated along with the degree of impact after their occurrence. Where 0 indicates no impact, 1 indicates a small degree of impact, 2 indicates a moderate degree of impact, and 3 indicates a large degree of impact. The level of harm of purchasing a lottery = impact level * frequency of occurrence; the 5 dimensions are summed up as the level of harm of purchasing a lottery, and the score ranges from 0 to 45 points. The higher the score, the deeper the level of harm encountered by buyers.

#### 3.2.2 Questionnaire on inappropriate publicity of sports lotteries

The Sports Lottery inappropriate Publicity Exposure Questionnaire developed by Shi Wenwen (11) was used to assess the frequency of sports lottery players’ exposure to Inappropriate publicity exposure information. The questionnaire consisted of four questions, such as “Exposure to reports and promotional information about the predictability of lottery numbers,” and was scored on a 5-point scale from 1 (never) to 5 (always). The higher the score, the more frequently Sports lottery players are exposed to inappropriate publicity.

#### 3.2.3 Obsessive passion questionnaire for lottery purchase

The Obsessive passion sub-questionnaire of the Betting Passion Questionnaire developed by Castelda,B.A. et al. (41) was used. The questionnaire was localized, and the model fit was good (RMSEA = 0.04, SRMR = 0.04, GFI = 0.98, CFI = 0.99, IFI = 0.99, NNFI = 0.97). Obsessive passion to buy lottery tickets consisted of 5 questions, such as “I have a hard time controlling my thoughts about buying lottery tickets.” It is scored on a 7-point scale from 1 (never) to 7 (always). The higher the score, the higher the level of obsessive passion to buy lottery tickets. The internal consistency reliability coefficient of the questionnaire in this study was 0.91.

#### 3.2.4 Questionnaire on lottery purchase behavior

The questionnaire on problem gambling behavior refers to the “Problem Gambling Questionnaire” compiled by Michael Auer and Neven Ricijas (Auer Michael et al. 2023). This questionnaire is used to measure the severity of problem gambling behavior. This questionnaire consists of 12 items, divided into 3 dimensions: gambling behavior (7 items), restriction (2 items), and communication (2 items). In this study, we only selected one dimension of gambling behavior and used it to develop a questionnaire for assessing problem lottery purchasing behavior. Using the 6 items in this dimension, such as “Having participated in more than 5 online gambling games within a week”, and scoring them on a 5-point scale, the higher the score, the more problematic the gambling behavior of the purchasers indicates. In this study, the Cronbach’s alpha coefficient of this questionnaire was 0.82, indicating that the questionnaire has good reliability.

#### 3.2.5 Responsible lottery buying beliefs questionnaire

The Responsible Purchase Scale for Sports Lottery Players compiled by WoodRTA (42) was used, including the Responsible Purchase Behavior subscale and the Responsible Purchase Belief subscale. The Responsible Lottery Purchasing Beliefs subscale was used in this study, which includes two dimensions: personal responsibility and sports lottery literacy. The personal responsibility dimension contains five questions, such as “It is my responsibility to purchase sports lottery tickets and spend only as much money as I can afford,” and the sports lottery literacy dimension contains three questions, such as “After I purchased sports lottery tickets and did not win the lottery, the next time I win the lottery, I will not be responsible for my behavior and my beliefs. After I buy a sports ticket and don’t win, the likelihood of winning increases the next time I win.” A 5-point scale is used, ranging from 1 (never agree) to 5 (strongly agree). Higher scores represent stronger health beliefs about responsible lottery purchases by sports bettors. The Cronbach’s coefficient of this questionnaire in this study was 0.894, indicating that the questionnaire has good reliability.

### 3.3 Data analysis

The Process plug-in of SPSS 27.0 was used to manage and analyze the data. Firstly, missing values in the data were replaced, and extremes were excluded using serial mean interpolation, while all data were then standardized. Second, demographic control variables were included, and correlation and regression analyses were performed for each variable. Bootstrap was used for mediation and moderated effect tests.

## 4 Findings

### 4.1 Common method bias test

Self-reported collection of data may lead to common method bias. For this reason, procedurally, this study was controlled by anonymous completion, reverse scoring of some questions, and polygraph questions; statistically, Harman’s one-way method was adopted for the common method bias test 43). The results show that the total number of factors with eigenroots greater than 1 is 14, and the cumulative variance explained by the first factor is 26.1, which is smaller than the critical criterion of 40%, indicating that there is no obvious common method bias in this study.

### 4.2 Overall level of sports lottery players’ harm in purchasing lottery tickets

#### 4.2.1 Differences in sports bettors’ lottery purchase hazards by gender

The results of the analysis of sports lottery players’ lottery purchase hazards and gender differences in each dimension are shown in **Table 2**. As can be seen from **Table 1**, the gender differences in the harms of buying lottery tickets and the dimensions have reached a level of significance (P<0.05).

**Table 2:** Descriptive statistics and analysis of variance of the harm of purchasing lottery tickets for sports lottery players of different genders.

Dimension	Male X ± S	Female X ± S	F	P
Physiological health	0.67 ± 1.37	0.39 ± 1.05	13.783	0.008
Negative mood	0.67 ± 1.45	0.43 ± 1.06	11.557	0.029
financial loss	0.79 ± 1.73	0.36 ± 1.02	35.205	<0.001
Relationships	0.60 ± 1.55	0.28 ± 0.95	20.829	0.004
Work/study	0.57 ± 1.46	0.24 ± 0.70	33.595	<0.001
Lottery playing harm	0.66 ± 1.39	0.34 ± 0.83	25.201	0.001

The difference between the total score and the scores of the dimensions of the harms of color purchasing by gender reaches a level of significance. That is, males suffered more from the hazards of purchasing lottery tickets than females. This may be because the number of male lottery players is larger. Compared with female lottery buyers who are interested in sports, male lottery buyers who are interested in sports will spend more time and energy on purchasing lottery tickets. They tend to do it more frequently and in larger quantities, and are also more likely to be negatively affected by lottery buying.

#### 4.2.2 Differences in the age of sports lottery players in terms of the hazards of purchasing lottery tickets

A one-way ANOVA was used to analyze the differences in the harm of Sports lottery players’ lottery purchases and the age differences in each dimension, and the results are shown in **Table 3**. As can be seen from **Table 3**, the differences in the total score of the harm of buying a lottery and the scores of each dimension on age did not reach a level of significance. (P>0.05).

**Table 3:** Descriptive statistics and ANOVA of the hazards of buying lottery tickets for Sports lottery players of different ages.

Dimension	Below 30 years	30–39 years	40–49 years	50–59 years	60 years and over	Overall	F	P
	X ± S	X ± S	X ± S	X ± S	X ± S	X ± S		
Physiological health	0.61 ± 1.39	0.45 ± 0.91	0.47 ± 1.12	0.68 ± 1.43	0.13 ± 0.33	0.54 ± 1.24	1.335	0.256
Negative mood	0.62 ± 1.42	0.38 ± 0.72	0.43 ± 1.03	0.76 ± 1.72	0.53 ± 1.44	0.55 ± 1.29	1.273	0.279
financial loss	0.61 ± 1.54	0.53 ± 1.20	0.55 ± 1.47	0.75 ± 1.59	0.49 ± 1.35	0.59 ± 1.46	0.27	0.898
Relationships	0.48 ± 1.40	0.32 ± 0.87	0.48 ± 1.51	0.57 ± 1.52	0.37 ± 1.47	0.45 ± 1.32	0.474	0.755
Work/study	0.46 ± 1.24	0.25 ± 0.58	0.51 ± 1.60	0.46 ± 1.12	0.29 ± 1.14	0.42 ± 1.18	0.924	0.449
Lottery playing harm	0.56 ± 1.28	0.39 ± 0.69	0.48 ± 1.25	0.65 ± 1.33	0.35 ± 0.10	0.51 ± 1.17	0.754	0.556

#### 4.2.3 Differences in the level of education of sports lottery players’ lottery purchase hazards

A one-way analysis of variance was used to compare the purchasing behaviors of lottery buyers and the differences in their educational levels. The analysis results are shown in **Table 4**. From **Table 4**, it can be seen that the differences in the overall score of lottery purchasing behavior and the scores of each dimension related to educational level did not reach a significant level (P < 0.05).

**Table 4:** Descriptive statistics and ANOVA of the harm of buying lottery tickets for Sports lottery players with different the level of education.

Dimension	Middle school and below	High school	College	Bachelor’s degree	Master’s degree and above	Overall	F	P
	X ± S	X ± S	X ± S	X ± S	X ± S	X ± S		
Physiological health	0.74 ± 1.58	0.55 ± 1.22	0.43 ± 0.93	0.52 ± 1.17	0.62 ± 1.62	0.54 ± 1.24	0.797	0.528
Negative mood	0.66 ± 1.51	0.47 ± 1.29	0.45 ± 1.10	0.56 ± 1.25	0.77 ± 1.50	0.55 ± 1.29	1.034	0.389
financial loss	0.78 ± 1.59	0.54 ± 1.36	0.63 ± 1.56	0.53 ± 1.36	0.56 ± 1.53	0.59 ± 1.46	0.408	0.803
Relationships	0.57 ± 1.71	0.46 ± 1.36	0.41 ± 1.00	0.44 ± 1.30	0.44 ± 1.50	0.45 ± 1.32	0.168	0.954
Work/study	0.60 ± 1.51	0.37 ± 1.11	0.43 ± 1.04	0.35 ± 1.04	0.45 ± 1.53	0.42 ± 1.18	0.567	0.687
Lottery playing harm	0.67 ± 1.47	0.48 ± 1.11	0.47 ± 1.04	0.48 ± 1.11	0.57 ± 1.38	0.51 ± 1.17	0.442	0.778

### 4.3 Analysis of the correlation between improper media exposure, obsessive enthusiasm for lotteries, problematic lottery betting behaviors, the hazards of lottery betting, and responsible betting concepts

The mean values, standard deviations and correlation coefficients of each variable are shown in **Table 5**. From the results of this table, it can be seen that improper promotion of lottery games is significantly positively correlated with forced excitement, problem lottery-buying behavior, and the hazards of lottery buying, and is significantly negatively correlated with the belief in responsible lottery buying; improper promotion is significantly positively correlated with forced excitement, problem lottery-buying behavior, and the hazards of lottery buying, and is significantly negatively correlated with the belief in responsible lottery buying; forced excitement, problem lottery-buying behavior and the hazards of lottery buying are all positively correlated with each other; the correlation analysis results show that gender, age and educational level are significantly correlated with the main variables of this study. Therefore, in the subsequent analysis, they are discussed as control variables. The above research results provide preliminary support for the verification of research hypotheses.

**Table 5:** Descriptive statistics and correlation analysis.

Variable	X ± S	Sex	Age	The level of education	Inappropriate publicity	Obsession	Problem lottery playing	Lottery playing harm	Responsible beliefs
Sex	1.46 ± 0.50	1							
Age	1.96 ± 1.19	-.182**	1						
The level of education	3.17 ± 1.19	.131**	-.466**	1					
Improper publicity	2.10 ± 1.06	-.125**	.154**	-.114**	1				
obsession	2.70 ± 1.45	-.148**	.212**	-.189**	.537**	1			
Problem lottery playing	1.80 ± 0.89	-.156**	.124**	-.102*	.701**	.664**	1		
Lottery playing harm	0.51 ± 1.17	-.133**	-0.014	-0.015	.323**	.423**	.461**	1	
Responsible Beliefs	3.80 ± 0.62	.149**	-.088*	.180**	-.253**	-.463**	-.417**	-.343**	1

*P ≤ 0.05, **P ≤ 0.01.

### 4.4 The impact of improper publicity exposure on the harm to lottery purchases, the individual mediating effect and chain mediating effect analysis of the obsessive enthusiasm for lottery purchases and problem lottery buying behaviors

According to Zhonglin et al. (44), Model 6 of the SPSS macro program PROCESS was used to test the mediating role of obsessive passion for color purchasing and problematic color purchasing behaviors between Improper publicity exposure and problematic color purchasing harms. After controlling for gender, age, and education, improper publicity significantly and positively predicted color-buying harms (β= 0.324, p<0.001); with the inclusion of two mediating variables, improper publicity significantly and positively predicted obsessive-obsessive passions (β= 0.507, p< 0.001) and problematic color-buying behavior (β= 0.487, p< 0.001); and obsessive-obsessive passions significantly and positively predicted problematic color-buying behavior (β= 0.408, p< 0.001) and significantly positively predicted lottery purchase harms (β= 0.224, p< 0.001); problematic lottery purchase behavior positively predicted lottery purchase harms (β= 0.32, p< 0.001), when improper publicity could not significantly negatively predict lottery purchase harms (B= -0.019, p=0.712). (See **Table 6**).

**Table 6:** Analysis of the chain-intermediary model of the harmful effects of improper promotion on lottery purchasing.

Mold	Regression equation		The overall number of fitted values			Significance of regression coefficients		
	Outcome variable	Predictor variable	R	R²	F	β	SE	t
One	Lottery playing harm	Sex	0.347	0.121	19.867	-0.106	0.040	-2.666**
		Age				-0.084	0.045	-1.877
		The level of education				0.000	0.044	-0.006
		inappropriate publicity				0.324	0.04	8.172***
Two	obsession	Sex	0.562	0.316	66.812	-0.063	0.035	-1.785
		Age				0.081	0.039	2.061*
		The level of education				-0.086	0.039	-2.220*
		inappropriate publicity				0.507	0.035	14.473***
Three	Problem lottery playing	Sex	0.783	0.613	183.199	-0.044	0.027	-1.639
		Age				-0.036	0.030	-1.199
		The level of education				0.024	0.029	0.802
		inappropriate publicity				0.487	0.031	15.833***
		obsession				0.408	0.031	13.03***
Four	Lottery playing harm	Sex	0.504	0.254	32.751	-0.069	0.037	-1.870
		Age				-0.103	0.041	-2.475**
		The level of education				0.024	0.041	0.591
		inappropriate publicity				-0.019	0.051	-0.37
		obsession				0.24	0.05	4.854***
		Problem lottery playing				0.32	0.058	5.531***

*P ≤ 0.05, **P ≤ 0.01, ***P ≤ 0.001.

The bootstrap program was used to conduct the mediation effect test with random sampling 5000 times. The 95% confidence intervals did not include 0. The results of the significance test for the mediation effect indicated: the total effect value was 0.325, the 95% confidence interval did not include 0, indicating a significant total effect. The direct effect value was -0.019, the 95% confidence interval included 0, and the direct effect was not significant. The total indirect effect value was 0.344, the 95% confidence interval did not include 0, indicating a significant mediation effect. Through a more detailed analysis, it was discovered that this regulatory effect involves three indirect influencing factors: indirect effect 1, improper promotion → forced passion → gambling harm (indirect effect = 0.122, SE = 0.032, 95% Cl = [0.064, 0.187]), the confidence interval did not include 0, indicating that the indirect effect of this path was significant; indirect effect 2, improper promotion → problem gambling behavior → gambling harm (indirect effect = 0.156, SE = 0.034, 95% Cl = [0.094, 0.228]), the confidence interval did not include 0, indicating that the indirect effect of this path was significant; indirect effect 3, improper promotion → forced passion → problem gambling behavior → gambling harm (indirect effect = 0.066, SE = 0.018, 95% Cl = [-0.037, -0.107]), the confidence interval did not include 0, indicating that the indirect effect of this chain relationship was significant.

The results showed that the direct effect was not significant, and this chain-mediated effect was mainly dominated by indirect effects. Among the indirect effects, the first mediation effect of forced passion accounted for 37.5%, the second mediation effect accounted for 48%, and the chain-mediated effect from forced passion to problem gambling behavior accounted for 20.3% (see **Table 7**).

**Table 7:** Decomposition table of direct, indirect and total effects of misleading promotions on lottery purchase risks.

Effect	Path	Boot’s standard error	Effect Value	Effect size	95% confidence interval	
					Lower limit	Upper limit
Indirect effect	X→M1→Y	0.032	0.122	35.5%	0.064	0.187
	X→M2→Y	0.034	0.156	45.3%	0.094	0.228
	X→M1→M2→Y	0.018	0.066	19.2%	0.037	0.107
Total indirect effect		0.056	0.344	100%	0.241	0.460

X1 represents improper publicity, X2 represents obsessive-obsessive passion, X3 represents problematic purchasing behavior, and X4 represents purchasing harm.

### 4.5 The moderating role of responsibility beliefs

#### 4.5.1 The moderating effect of responsible betting beliefs in the mediating model that exposes the impact of improper promotion on the harm of lottery playing

The moderating effect of this chain was tested in this study using model 92 of the SPSS macro program PROCESS plug-in. After placing responsibility beliefs into the chain mediation, the product term of improper publicity and responsibility beliefs was a significant negative predictor of obsessive passion.(β=-0.254, P<0.001), indicating that in the pathway of improper publicity on obsessive passion, responsibility purchasing beliefs were able to modulate the positive predictive effect of improper publicity on obsessive passion; the negative predictive effect of the product term of improper publicity and responsibility beliefs on problematic lottery purchasing behaviors was significant (β=-0.351, P<0.001), indicating that responsibility purchasing beliefs were able to modulate the positive predictive effect of improper publicity on The negative predictive effect of the product term of the problem lottery playing and responsibility beliefs on the harm of lottery purchase was significant (β=-0.39, P<0.001), indicating that the responsibility beliefs were able to modulate the predictive effect of the problem lottery playing on the harm of lottery purchase. (See **Table 8**).

**Table 8:** Analysis of the chain mediation model of responsibility belief in the moderating effect of improper advertising on the harmful impact on lottery purchasing.

Model	Regression equation		The overall number of fitted values			Significance of regression coefficients		
	Outcome variable	Predictor variable	R	R²	F	β	SE	t
One	Obsessive passion	Sex	0.662	0.439	75.207	-0.032	0.032	-0.999
		Age				0.086	0.036	2.402*
		The level of education				-0.045	0.036	-1.266
		inappropriate publicity				1.365	0.2	6.813***
		Responsible Beliefs				-0.576	0.053	-10.841***
		Misadvocacy* Responsibility beliefs				-0.254	0.054	-4.732***
Two	Problem lottery playing	Sex	0.819	0.67	146.002	-0.043	0.025	-1.722
		Age				-0.033	0.028	-1.184
		The level of education				0.02	0.028	0.721
		inappropriate publicity				1.801	0.173	10.4***
		Obsessive passion				0.409	0.2	2.041*
		Responsibility Beliefs				2.041	0.048	-6.664***
		Undue Propaganda * Responsibility Beliefs				-0.351	0.046	-7.706***
		Obsessive passion * Belief in Responsibility				-0.032	0.056	-0.58
Three	Problem lottery playing	Sex	0.554	0.307	25.372	-0.082	0.036	-2.272*
		Age				-0.101	0.04	-2.519*
		The level of education				0.033	0.04	0.814
		Improper publicity				-0.055	0.325	-0.169
		Forced passion				0.579	0.328	1.763
		Problem lottery playing				1.616	0.426	3.79***
		Responsible Beliefs				-0.381	0.072	-5.286***
		Misrepresentation*Responsibility Beliefs				0.017	0.083	0.203	
		Obsessive passion* Responsible Beliefs				-0.108	0.091	-1.18
	Problem lottery playing* Responsibility Beliefs				-0.39	0.117	-3.33***

*P ≤ 0.05, ***P ≤ 0.001.

Similarly, the simple slope test method was adopted to further conduct an in-depth analysis of the regulatory mechanism of the sense of responsibility. The beliefs about responsible lottery purchasing were divided into high and low groups according to plus or minus one standard deviation. The impacts of improper publicity on obsessive passion and problem lottery purchasing behavior, as well as the impact of problem lottery purchasing behavior on the harm of lottery purchasing, were examined under different levels of beliefs about responsible lottery purchasing. The results are shown in **Table 8**. In the group with low beliefs about responsible lottery purchasing (M - 1SD), the positive predictive effect of improper publicity on obsessive passion was significant (β = 0.54, p < 0.001), the positive predictive effect of improper publicity on problem lottery purchasing behavior was significant (β = 0.66, p < 0.001), and the predictive effect of problem lottery purchasing behavior on the harm of lottery purchasing was significant (β = 0.349, p < 0.001). In the group with high beliefs about responsible lottery purchasing (M + 1SD), the predictive effect of improper publicity on obsessive passion was significant (β = 0.222, p < 0.001), the predictive effect of improper publicity on problem lottery purchasing behavior was significant (β = 0.222, p < 0.001), while the predictive effect of problem lottery purchasing behavior on the harm of lottery purchasing was not significant (β = -0.138, p > 0.05). This indicates that the beliefs about responsible lottery purchasing buffered the impacts of improper publicity on obsessive passion and problem lottery purchasing behavior, as well as the impact of problem lottery purchasing behavior on the harm of lottery purchasing. (See **Table 9**).

**Table 9:** Test of the moderated chain mediation model.

Outcome variables	Predictor variables	Responsibility purchase beliefs	Efficacy values	Boot standard error	Boot CI lower bound	Boot CI upper bound
		3.25	0.54	0.04	0.461	0.618
Obsessive passion	Undue publicity	3.75	0.413	0.033	0.348	0.477
		4.5	0.222	0.055	0.115	0.329
		3.25	0.66	0.036	0.589	0.732
Problem lottery playing	Improper publicity exposure	3.75	0.485	0.028	0.429	0.541
		4.5	0.222	0.044	0.134	0.309
		3.25	0.349	0.074	0.205	0.494
Hazards of Lottery Purchasing	Problem lottery playing	3.75	0.154	0.063	0.031	0.278
		4.5	-0.138	0.121	-0.376	0.1

**Figure 2:**
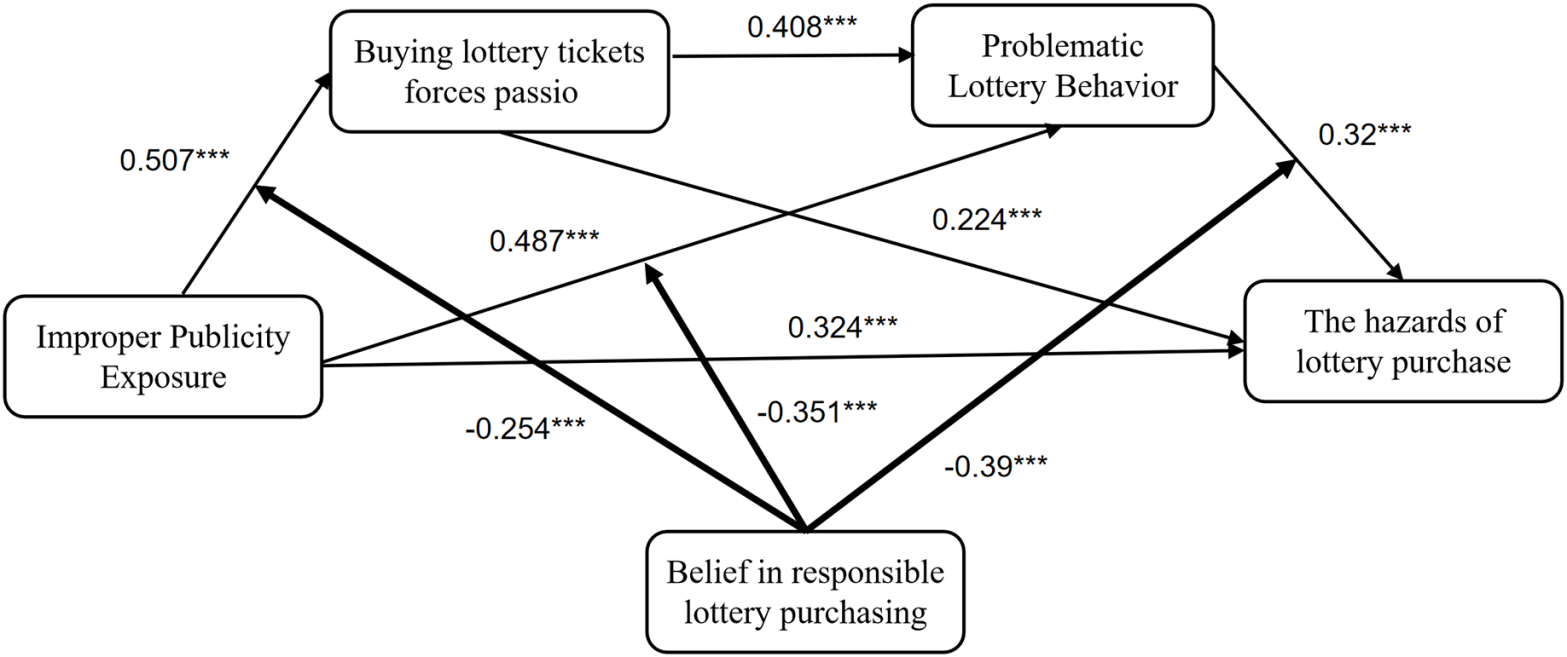
The model results of studying the relationships among variables. ***P ≤ 0.001.

The asterisks above the right side of the numbers in **Figure 2** indicate significance. One asterisk indicates a p-value < 0.05, two asterisks indicate a p-value < 0.01, and three asterisks indicate a p-value < 0.001.

## 5 Research discussion

### 5.1 The level of harm caused by lottery purchasing among sports lottery players

The findings of this study indicate that the harms inflicted on sports lottery players due to lottery purchasing, in descending order of severity, are manifested as financial loss hazards, adverse emotional hazards, physical health hazards, interpersonal relationship hazards, and work or study hazards. Male players suffer greater harm from lottery purchasing compared to female players, which is consistent with the results of previous studies. On one hand, this may be attributed to the fact that, in contrast to female lottery players, male lottery players tend to purchase lottery tickets more frequently and in larger quantities, making them more susceptible to the adverse impacts brought about by lottery purchasing. On the other hand, during the process of lottery purchasing, men are more prone to taking risks and engaging in competition and are more likely to be trapped in the dilemma of problematic lottery purchasing and thus suffer from the associated harms. In contrast, female sports lottery players are more cautious and conservative, and they are more capable of controlling their lottery purchasing behavior.

### 5.2 The impact of improper publicity of sports lotteries on the harms of lottery purchasing

This study indicates that improper promotion of lottery games can have a positive predictive effect on the harm caused by lottery purchases. Hypothesis H1 has thus been verified. Firstly, improper publicity of sports lotteries can directly lead to the emergence of speculative mindsets, Negative mood, and financial hazards among sports lottery players. For example, as Erika Langham discovered through interviews, excessive gambling or gambling addiction can mainly cause emotional harm to individuals. This is manifested in the lack of control over behavior or environment by the gamblers, which leads to feelings of insecurity and shame. There is both a sense of powerlessness due to being unable to manage gambling behavior, and a sense of despair in trying to recover losses. Hu Yue’s (45) research found that irrational gambling by sports bettors can cause emotional harm. For instance, feeling distressed because of not winning, regret and pain due to excessive energy, money and time investment in gambling, and losing money due to impulsive behavior during gambling. All of these bring unpleasant experiences, which in turn cause Negative mood in sports bettors. The dissemination of improper sports lottery information through various communication means and channels reinforces the unhealthy psychological states of sports lottery players, thereby increasing the risks associated with lottery purchasing (15)and giving rise to the harms of lottery purchasing. These harms are not confined to the individual lottery purchasers; they can also spread to significant others in their lives (46)and even affect the entire society through financial problems and criminal activities, ultimately resulting in a decline in the overall social well-being and quality of life (47).

### 5.3 The complete mediating role of obsessive passion and problem lottery purchasing behavior in the impact of improper publicity on the harms of lottery purchasing

This study demonstrates that after incorporating obsessive passion and problematic lottery purchasing behavior, the predictive effect of improper publicity of sports lotteries on the harms of lottery purchasing is no longer significant. Obsessive passion and problematic lottery purchasing behavior play a chained mediating role in the process where improper publicity of sports lotteries leads to the harms of lottery purchasing. Hypotheses H2, H3, and H4 are thus validated.

Firstly, obsessive passion acts as a mediator between the improper publicity of sports lotteries and the harms of lottery purchasing. Firstly, according to the dual model of passion, obsessive passion mostly stems from the excitement obtained during an activity, which leads to the inability to control one’s behavior. Improper publicity of sports lotteries (such as marketing forms like brand effects, financial incentives, and odds advertisements) can arouse a strong emotional arousal among lottery players, making it difficult for them to control the consequences of their actions, and thus leading to irrational psychological states and excessive economic expenditures (26). Secondly, according to the stimulus-response theory, gambling stimuli (such as having won a lottery or hitting a big prize) will trigger psychological responses (gambling consumption intentions and consumption emotions) among lottery players. Especially, sports lottery players who have a high sensitivity to rewards and tend to seek immediate reward effects are more vulnerable to the influence of information about winning big prizes or having already won, thus giving rise to the harms of lottery purchasing (48). Thirdly, improper publicity of sports lotteries can stimulate the positive emotions of lottery players through false and exaggerated information, thereby increasing their willingness to purchase lottery tickets. For example, improper publicity of sports lotteries can make lottery players feel that lottery purchasing is “fun” or that they “have won” (49), leading to the formation of wrong beliefs among lottery players, such as cognitive distortions and illusions of control, which in turn exacerbates the severity of problematic lottery purchasing and brings about the harms of lottery purchasing (37).

Secondly, problematic lottery purchasing behavior acts as a mediator between improper publicity and the harms of lottery purchasing. Firstly, based on the mere exposure effect, lottery players develop a habit of perceiving and processing lottery advertisements due to frequent exposure to them, and inadvertently strengthen the conditioned responses of lottery purchasing intentions, impulses, or desires, resulting in the loss of control over emotions and behaviors along with lottery-related associations (50). Studies have shown (51) that scenes that are unrelated to gambling but contain exciting elements (such as watching a football match) can trigger memories among problematic gamblers and arouse a certain degree of gambling impulse in them. Some studies have also found that exposure to gambling advertisements can directly lead to an increase in the willingness and behavior of gambling (52). Secondly, based on Bandura’s social learning theory, during the process of lottery purchasing, lottery players may actively or passively learn various lottery purchasing information, behaviors, skills, or response patterns of others, which will have a significant impact on their own lottery purchasing beliefs and behaviors. The display of positive outcomes of others’ lottery purchasing in improper lottery publicity will hurt the attitudes and behaviors of sports lottery players towards lottery purchasing, increasing the risk of problematic lottery purchasing (SunYue, 2022). Studies have shown that when adolescents observe gambling behaviors among their peers, they will imitate them, thus promoting the development of their future problematic gambling (53).

Thirdly, obsessive passion and problematic lottery purchasing behavior play a chained mediating role in the impact of improper publicity of sports lotteries on the harms of lottery purchasing. Firstly, publicity information often exerts an influence on individuals by stimulating their emotional and cognitive needs, thereby inducing an irrational state, which leads to irrational decision-making during lottery purchasing (54). Lottery purchasing decisions driven by such a highly emotional state are often personalized and action/result-oriented and are ultimately likely to give rise to lottery purchasing problems and bring about the harms of lottery purchasing (55). Secondly, based on the priming effect theory, improper publicity information in sports lotteries will establish connections with individuals’ knowledge and experiences related to lottery purchasing in their past experiences or memories, triggering obsessive passion for lottery purchasing, giving rise to problematic lottery purchasing behavior, and accompanied by harms such as emotional disorders, psychological distress, and increased debt burdens (35). A study on Swedish gamblers reported that most gamblers believed that the exaggerated marketing of online sports betting made it difficult for them to resist the gambling impulse, and led to a loss of control over their gambling behavior, causing their gambling behavior to exceed expectations and resulting in worsened consequences, such as individuals who had refused to gamble experiencing repeat purchasing behavior (56).

### 5.4 The moderating role of the belief in responsible lottery purchasing

In addition, this study has found that the sense of responsibility not only moderates the impact of improper publicity of sports lotteries on the obsessive passion for lottery purchasing but also moderates the impact of improper publicity of sports lotteries on problematic lottery purchasing behavior. Hypotheses H5, H6, and H7 are thus validated.

This study has found that the belief in responsible lottery purchasing can moderate the impact of improper publicity on obsessive passion. Firstly, compared with sports lottery players with low levels of the sense of responsibility, improper publicity generates a lower level of obsessive passion and poses a smaller risk of problematic lottery purchasing among sports lottery players with high levels of the sense of responsibility. This is consistent with the integrated model of “risk-protection” factors for lottery purchasing health (11) that is, when a risk factor (improper publicity) affects obsessive passion, an individual’s protective factor (the belief in responsible lottery purchasing) interacts with it, buffering its negative impact. Secondly, improper publicity of sports lotteries can directly trigger intense emotions among lottery players during the lottery purchasing process, such as impulsive lottery purchasing (especially among problematic lottery players (37). In contrast, sports lottery players with the belief in responsible lottery purchasing show fewer emotional manifestations and can make rational decisions during the lottery purchasing process (39). Therefore, an increase in the belief in responsible lottery purchasing among sports lottery players can reduce the obsessive passion for lottery purchasing caused by exposure to improper publicity of sports lotteries.

This study also shows that the belief in responsible lottery purchasing can moderate the impact of improper publicity of sports lotteries on problematic lottery purchasing behavior. The reasons are as follows: The belief in responsible lottery purchasing incorporates the expectations of social values and requires gamblers to fulfill their obligations of responsible lottery purchasing (57). Gamblers with the belief in responsible lottery purchasing have a higher level of attitude towards lottery purchasing, subjective norms, and perceived behavioral control. During the lottery purchasing process, they tend to adopt some protective behaviors (such as pre-commitment, money setting, time limit, etc.), and the impact of improper publicity information on them is relatively small or infrequent (58). Some studies have shown that there is a positive correlation between an individual’s level of belief in responsibility and a lower risk of problematic gambling. Secondly, individuals should control their behaviors through rationality, self-restraint, and informed choice to avoid the weakening of the inhibition of pathological gambling caused by a loss of control (59). The Rienzo model (60) points out that the ultimate decision-making power in gambling still lies with the individual. Individuals with correct gambling beliefs can take actions to limit their gambling expenditures within an affordable range to avoid gambling-related harm.

In addition, compared with sports lottery players with low levels of belief in responsible lottery purchasing, problematic lottery purchasing behavior does not cause harm to lottery purchasing for sports lottery players with high levels of belief in responsible lottery purchasing. This indicates that even if problematic lottery purchasing behavior occurs, maintaining a high level of belief in responsible lottery purchasing can, to a certain extent, alleviate the harms of lottery purchasing. Firstly, the problematic gambling behavior of sports lottery players is likely to be triggered by the beautiful anticipation of winning the lottery and making a fortune, which may be related to emotional motivational decision-making. The belief in responsible lottery purchasing can offset the impacts of external unfavorable information, the lottery players’ experiential thinking, etc., on their gambling beliefs and behaviors (39), thereby reducing the harms of gambling. Secondly, lottery players with the belief in responsible lottery purchasing can control their lottery purchasing habits through their own will and ability, without being affected by negative factors and irrational thoughts (40). At the same time, they will make lottery purchasing decisions based on sufficient, timely, and accurate information (that is, informed choice) and apply self-regulatory strategies or adopt external strategies (for example, pre-commitment) to participate in gambling within an affordable range (9), so the occurrence of problematic gambling behavior is less likely, avoiding the generation of gambling harms. Studies have found (61) that whether one has the belief in responsible lottery purchasing is the fundamental difference between individuals who can control their gambling behavior (responsible gamblers) and those who cannot (disordered/pathological gamblers). Therefore, the belief in responsible lottery purchasing can moderate the effect of problematic lottery purchasing behavior on the harms of lottery purchasing.

In conclusion, considering the predictive effect of improper publicity of sports lotteries on the harms of lottery purchasing and the moderating role of the belief in responsible lottery purchasing, initiatives can be taken from multiple aspects. Firstly, lottery information should be rationally publicized in the content of publicity advertisements. When formulating promotional information about lottery prizes, the content in the publicity information that is likely to arouse the obsessive passion and lottery purchasing behavior of lottery players (such as the ease of winning and winning big prizes) should be reduced, and the content that can arouse the harmonious passion of lottery players, such as emphasizing the social responsibility and public welfare attributes of lotteries, should be increased. Secondly, attention should be paid to enhancing the level of the belief in responsible lottery purchasing among sports lottery players. Although there are currently some relevant studies on responsible lottery purchasing, most of them focus on dividing the responsibilities of the lottery industry, the government, and society, as well as investigating whether lottery players adopt responsible lottery purchasing strategies; there is still a lack of specific guidance on the belief in responsible lottery purchasing and the cultivation approaches. Therefore, the belief in responsible lottery purchasing among sports lottery players can be cultivated through public education and publicity initiative activities, such as providing baseline information on the knowledge, values, and beliefs related to responsible lottery purchasing, and developing and improving the public’s awareness of lottery purchasing responsibility (62), to increase the public’s understanding and awareness of rational and responsible lottery purchasing.

## 6 Research conclusions

(1) Exposure to improper publicity of sports lotteries will cause harm to sports lottery players due to lottery purchasing. These harms, in descending order of severity, are financial loss hazards, Negative moodal hazards, physical health hazards, interpersonal relationship hazards, and work/study hazards.

(2) Improper publicity of sports lotteries can increase the harms of lottery purchasing for sports lottery players through three pathways: Firstly, it can arouse the obsessive passion for lottery purchasing among sports lottery players, which in turn leads to the harms of lottery purchasing suffered by sports lottery players. Secondly, it can increase the risk of problematic lottery purchasing among sports lottery players, thereby exacerbating the harms of lottery purchasing. Thirdly, it can increase the obsessive passion for lottery purchasing among sports lottery players, leading to an intensification of the risk of problematic lottery purchasing and thus giving rise to the harms of lottery purchasing.

(3) The belief in responsible lottery purchasing can mitigate the harms of lottery purchasing. On the one hand, the belief in responsible lottery purchasing can alleviate the obsessive passion for lottery purchasing and the risk of problematic lottery purchasing caused by improper publicity of sports lotteries. On the other hand, the belief in responsible lottery purchasing can mitigate the harms of lottery purchasing brought about by the risk of problematic lottery purchasing.

## Data Availability

The raw data supporting the conclusions of this article will be made available by the authors, without undue reservation.
