# Supplementary material for: The impact of improper promotion of sports lottery on the harm of purchasing sports lottery: an intermediary in a regulatory chain
Source: Front Psychiatry. 2026 Feb 4;16:1670697. doi: 10.3389/fpsyt.2025.1670697 (PMC12913116; doi:10.3389/fpsyt.2025.1670697)
Supplement: Supplementary file 1 [file Table1.docx]

Supplementary Material

The impact of improper promotion of sports lottery on the harm of purchasing sports lottery: An intermediary with a regulatory chain

# Supplementary Tables

## Table 1

Table 1 List of basic information of survey respondents (N=586)

| Category | Attribute | Number of persons | Percentage (%) |
| --- | --- | --- | --- |
| Gender | Male | 317 | 54.2% |
|  | Female | 268 | 45.8% |
| Age | Below 30 years old | 298 | 50.9% |
|  | 30-44 years old | 171 | 29.2% |
|  | 45-59 years old | 92 | 15.7% |
|  | 60 years old and above | 25 | 4.3% |
| Educational attainment | Junior high school or below | 65 | 10.6% |
|  | High school or vocational high school | 112 | 19.1% |
|  | College | 154 | 26.3% |
|  | Bachelor's degree | 181 | 30.9% |
|  | Graduate students and above | 77 | 13.1% |
| Years of purchasing lottery tickets | 1 year and below | 206 | 35.2% |
|  | 2~3 years | 173 | 29.5% |
|  | 4~6 years | 93 | 15.9% |
|  | 7~10 years | 57 | 9.7% |
|  | More than 10 years | 57 | 9.7% |
| Monthly Personal Income | $2000 and below | 125 | 21.3% |
|  | 2001~4000 Yuan | 154 | 26.3% |
|  | 4001~6000 Yuan | 157 | 26.8% |
|  | 6001~8000 Yuan | 63 | 10.8% |
|  | Above 8000 yuan | 87 | 14.8% |

## Table 7

Table 7 Breakdown of direct, indirect, and total effects of Inappropriate publicity exposure exposure on the harm of lottery purchase

| mold | Regression equation | |  | The overall number of fitted values | | |  | Significance of regression coefficients | | |
| --- | --- | --- | --- | --- | --- | --- | --- | --- | --- | --- |
|  | Outcome variable | Predictor variable |  | R | R² | F |  | β | SE | t |
| one | Hazards of Lottery Purchase | Sex |  | 0.347 | 0.121 | 19.867 |  | -0.106 | 0.040 | -2.666** |
|  |  | Age |  |  |  |  |  | -0.084 | 0.045 | -1.877 |
|  |  | Academic qualifications |  |  |  |  |  | 0.000 | 0.044 | -0.006 |
|  |  | Misinformation |  |  |  |  |  | 0.324 | 0.04 | 8.172*** |
|  |  |  |  |  |  |  |  |  |  |  |
| two | obsession | Sex |  | 0.562 | 0.316 | 66.812 |  | -0.063 | 0.035 | -1.785 |
|  |  | Age |  |  |  |  |  | 0.081 | 0.039 | 2.061* |
|  |  | Academic qualifications |  |  |  |  |  | -0.086 | 0.039 | -2.220* |
|  |  | Misinformation |  |  |  |  |  | 0.507 | 0.035 | 14.473*** |
|  |  |  |  |  |  |  |  |  |  |  |
| three | Problematic Lottery Behavior | Sex |  | 0.783 | 0.613 | 183.199 |  | -0.044 | 0.027 | -1.639 |
|  |  | Age |  |  |  |  |  | -0.036 | 0.030 | -1.199 |
|  |  | Academic qualifications |  |  |  |  |  | 0.024 | 0.029 | 0.802 |
|  |  | Misinformation |  |  |  |  |  | 0.487 | 0.031 | 15.833*** |
|  |  | obsession |  |  |  |  |  | 0.408 | 0.031 | 13.03*** |
|  |  |  |  |  |  |  |  |  |  |  |
| four | Hazards of Lottery Purchase | Sex |  | 0.504 | 0.254 | 32.751 |  | -0.069 | 0.037 | -1.870 |
|  |  | Age |  |  |  |  |  | -0.103 | 0.041 | -2.475** |
|  |  | Academic qualifications |  |  |  |  |  | 0.024 | 0.041 | 0.591 |
|  |  | Misinformation |  |  |  |  |  | -0.019 | 0.051 | -0.37 |
|  |  | obsession |  |  |  |  |  | 0.24 | 0.05 | 4.854*** |
|  |  | Problematic Lottery Behavior |  |  |  |  |  | 0.32 | 0.058 | 5.531*** |

## Table 9

Table 9 Chain mediation model analysis of responsibility beliefs in moderating the influence of inappropriate publicity on lottery purchase harms

| Model | Regression equation | |  | The overall number of fitted values | | |  | Significance of regression coefficients | | |
| --- | --- | --- | --- | --- | --- | --- | --- | --- | --- | --- |
|  | Outcome variable | Predictor variable |  | R | R² | F |  | β | SE | t |
| One | Compulsive Passion | Sex |  | 0.662 | 0.439 | 75.207 |  | -0.032 | 0.032 | -0.999 |
|  |  | Age |  |  |  |  |  | 0.086 | 0.036 | 2.402* |
|  |  | Academic qualifications |  |  |  |  |  | -0.045 | 0.036 | -1.266 |
|  |  | Misinformation |  |  |  |  |  | 1.365 | 0.2 | 6.813*** |
|  |  | Responsible Beliefs |  |  |  |  |  | -0.576 | 0.053 | -10.841*** |
|  |  | Misadvocacy* Responsibility beliefs |  |  |  |  |  | -0.254 | 0.054 | -4.732*** |
|  |  |  |  |  |  |  |  |  |  |  |
| two | Problematic Lottery Behavior | Sex |  | 0.819 | 0.67 | 146.002 |  | -0.043 | 0.025 | -1.722 |
|  |  | Age |  |  |  |  |  | -0.033 | 0.028 | -1.184 |
|  |  | Academic qualifications |  |  |  |  |  | 0.02 | 0.028 | 0.721 |
|  |  | Misinformation |  |  |  |  |  | 1.801 | 0.173 | 10.4*** |
|  |  | Compulsive Passion |  |  |  |  |  | 0.409 | 0.2 | 2.041* |
|  |  | Responsibility Beliefs |  |  |  |  |  | 2.041 | 0.048 | -6.664*** |
|  |  | Undue Propaganda * Responsibility Beliefs |  |  |  |  |  | -0.351 | 0.046 | -7.706*** |
|  |  | Compulsive Passion * Belief in Responsibility |  |  |  |  |  | -0.032 | 0.056 | -0.58 |
|  |  |  |  |  |  |  |  |  |  |  |
| three | Problematic Lottery Behavior | Sex |  | 0.554 | 0.307 | 25.372 |  | -0.082 | 0.036 | -2.272* |
|  |  | Age |  |  |  |  |  | -0.101 | 0.04 | -2.519* |
|  |  | Academic qualifications |  |  |  |  |  | 0.033 | 0.04 | 0.814 |
|  |  | Inappropriate publicity |  |  |  |  |  | -0.055 | 0.325 | -0.169 |
|  |  | Forced passion |  |  |  |  |  | 0.579 | 0.328 | 1.763 |
|  |  | Problematic Lottery Behavior |  |  |  |  |  | 1.616 | 0.426 | 3.79*** |
|  |  | Responsible Beliefs |  |  |  |  |  | -0.381 | 0.072 | -5.286*** |
|  |  | Misrepresentation*Responsibility Beliefs |  |  |  |  |  | 0.017 | 0.083 | 0.203 |
|  |  | Compulsive Passion* Responsible Beliefs |  |  |  |  |  | -0.108 | 0.091 | -1.18 |
|  |  | Problematic Lottery Behavior* Responsibility Beliefs |  |  |  |  |  | -0.39 | 0.117 | -3.33*** |
